# The bishop and the actress

**DOI:** 10.1186/2041-2223-3-27

**Published:** 2012-12-24

**Authors:** Mark A Jobling

**Affiliations:** 1 Department of Genetics, University of Leicester, University Road, Leicester, LE1 7RH, UK

## 

It was J.B.S. Haldane who once irreverently declared that ‘even the Archbishop of Canterbury is 65% water’. As it happens, his grace is also about 1% DNA, a fact that some elevated clergy are apparently reluctant to acknowledge. One bishop opined to a colleague of mine: ‘Of course, DNA doesn’t actually *exist*, does it…’ He seemed to regard the double helix like the Holy Spirit - a somewhat intangible and metaphorical thing. Since many bishops are members of the House of Lords of the UK Parliament and play a role in passing legislation, including rulings about genetics, this is a disturbing attitude. My colleague’s decisive response was to corral a bevy of bishops (probably not the standard collective noun, but happily alliterative nonetheless) into the laboratory to prepare some very tangible DNA from bananas.

DNA may be real enough, but it has increasingly come to stand as a metaphor. For example, the actress Lesley Sharp tells us that ‘there is something quintessentially northern in my DNA’; Sir Alex Ferguson apparently believes that ‘late goals are in Manchester United’s DNA’, UK Foreign Secretary William Hague thinks that human rights ‘are part of our national DNA’; and an article about crisps avers that ‘a love of the potato is hard-wired into our gastronomic DNA’. How this humble biomolecule has come to symbolize geography, football, civil liberties and snack-foods is a curious business.

In these metaphors, DNA represents Nature - the pack of cards we are dealt - as opposed to Nurture, and a major determinant of our characteristics. If that’s really the way it is, then a complete genome sequence should have an awful lot to say about the person who carries it. Next-generation sequencing is providing unprecedented insights, and the recent publication of 1,092 such sequences [1] is a major step towards a better understanding.

The importance of Nature in a complex trait or disease is measured by its heritability, while the Nurture part is the environment. The well-rehearsed problem with studies of the association of variants in multiple genes with phenotypes is that of ‘missing heritability’. For example, the classical quantitative trait of adult height is approximately 80% heritable, and because the trait has been recorded incidentally as part of the phenotype in many genomewide association studies (GWASs), it has been possible to assemble very large sample sets for meta-analysis. A study of >180,000 individuals [2] identified hundreds of common genetic variants influencing height in at least 180 loci, and yet together these explain only a pitiful 10% of the variation in the trait. Modeling based on the distribution of variant effect sizes and the power to detect them suggested that a sample size of half a million people would reveal common variants explaining an underwhelming <16% of the variation. In response to this problem, practitioners of the subtle art of the GWAS have adjusted their mission statements: instead of trying to explain the variation in traits through countless loci with infinitesimal effects, they are after the biological pathways that play roles in the pathology of disease, and will ultimately offer possible drug targets. Well…we certainly all hope so.

But where has all the heritability gone? The common-disease-common-variant (CDCV) hypothesis [3] had suggested that common alleles (≥1% frequency) with moderate or small effects were responsible for the effects of genes on phenotypes. Just as it is now impossible to find anyone who voted for Margaret Thatcher in the 1980s, even though she won a record-breaking three terms in Parliament, it is now difficult to find anyone who admits to have believed in the CDCV idea, despite its evident popularity at the time. Copy-number variants were in the frame as alternative suspects for a while, because these were expected to be poorly tagged by the SNPs typed in GWASs; however, this didn’t work out. Much attention has since shifted [4] to putative rare variants (<1%) with large effects, which is where next-generation sequencing comes in, and this certainly provides a good justification for very large grant applications. Eric Lander, one of the architects of the CDCV hypothesis, is now placing his faith in gene-gene interactions (implicitly ignored in most models of complex disease), and believes that ‘missing’ is really ‘phantom’ [5]. For example, he suggests that 80% of the missing heritability of Crohn’s disease could be explained by gene-gene interactions, if the disease involves interactions among three biochemical pathways.

Is there anything else? One slippery customer lurks in the shadows - epigenetics. The ‘epi’ part means ‘above’, and the term covers modifications to DNA that do not affect its nucleotide sequence, but can influence gene expression. Most often included in this category are methylation of cytosines in DNA itself, and chemical modifications of the histones around which the DNA is wrapped, though there are additional potential players, including RNA-induced gene silencing and other mechanisms involved in establishing and maintaining chromatin structure and nuclear matrix attachment.

Clearly, if epigenetics is to explain any of the ‘missing heritability’, some aspect of the epigenetic marking of the genome must itself be heritable. The best-studied modification is DNA methylation, because it’s the most easily measured, and the conventional wisdom is that this is erased and reprogrammed during gametogenesis. This makes sense because the methylation patterns of different cell-types differ, and if the zygote is to be able to give rise to all of these cell-types, it needs to start from scratch each generation, establishing methylation patterns anew during development. However, although the results of comparative studies of specific loci in human mono- and dizygotic twins are complex, overall they do indicate some heritability of DNA methylation [6].

Other lines of evidence certainly indicate that there is something heritable to explain. Some phenomena, such as genome instability, can echo through several generations: irradiate mice with ionizing radiation, or treat them with anticancer drugs [7], and their grandchildren, as well as their children, manifest genome instability. This transgenerational memory of a genomic insult has to be mediated via some epigenetic phenomenon, albeit an undefined one.

Perhaps more than its possible contribution to heritable effects, epigenetics is becoming the poster child of real-time modification of the output of our genomes. Many animal studies show that early life exposures through maternal diet can lead to changes in methylation or histone modification. More newsworthy, however, has been the report of methylation-induced gene expression changes in humans following acute exercise [8].

The existence of something beyond the genome that can control gene expression, can be modified by life experiences, and shows effects across generations has attracted the attention of a number of interesting communities. A little Googling reveals a buzz among intelligent-design freaks, homeopaths, neo-Lamarckians and diet gurus. One company selling dietary supplements apparently designed to modify epigenetic profiles proclaims that ‘It’s Time To Kick Obesity's Epigenetic Butt!’, and ‘Epigenetics Means: Its Never To Late’ (sic). Among the religious, there is some discussion of theological problems, including the long-term epigenetic effects of the Fall of Adam, and Moses’ cheerful message that God will visit the iniquity of the fathers on the children to the third and the fourth generations of those who hate him. We await the bishops’ epipronouncements with interest.
